# Success in Vaccination Efforts of Vulnerable Populations in the WHO/European Region: Focus on Prisons

**DOI:** 10.3389/fpubh.2021.738422

**Published:** 2021-10-05

**Authors:** Filipa Alves da Costa, Yanina Andersen, Carina Ferreira-Borges

**Affiliations:** Alcohol, Illicit Drugs & Prison Health Programme, WHO European Office for Prevention and Control of Noncommunicable Diseases, Moscow, Russia

**Keywords:** prisons, vulnerable populations, vaccination, COVID-19, Europe

COVID-19 has brought the world's attention to the fragility of mankind in general, highlighting particular risk for vulnerable populations from being hit and having more severe outcomes, including death. The concept of vulnerability generates considerable disagreement, ranging from considerations of social justice factors to the impact on health (1). There is, however, consensus that vulnerability is not a static characteristic but a situational descriptor that may change over time. For example, the restrictions measures imposed because of COVID-19 have led some people to situations of vulnerability, when for instance becoming unable to pay for their house due to job losses. The same concept applies to people who, at a certain moment in life, become deprived of liberty.

The increased susceptibility of people living in detention to contracting infectious diseases is well-established, and most measures implemented focus on preventing the virus to enter prison walls. There is no doubt that the potential impact of COVID-19 in the prison population is higher because access to healthcare is often suboptimal and because the burden of underlying health conditions is higher. In March 2021, Neufeld et al., called for the need for prisons to be included in global and national vaccination efforts against COVID-19 (2). This message was strengthened by an advocacy brief launched in June by the WHO Health in Prisons Programme (WHO-HIPP), developed in collaboration with the United Nations Office on Drugs and Crime and Penal Reform International (3). The announced intention of this brief was to advocate for the reduction of inequalities in healthcare provision for all vulnerable groups to ensure full attainment of universal health coverage by leaving no one behind, aligned with the bold intentions of the WHO European Programme of Work 2020–2025 (4). In this follow-up piece we further highlight the notable efforts of selected Member States to ensure COVID-19 vaccination coverage.

WHO-HIPP has developed a surveillance system for places of detention that relies on voluntary reports from Member States (5). In February 2021 this reporting system was adapted to include information on vaccines administered to people in prison, staff and health workers in the criminal justice system.

Since March, all healthcare workers in Spanish prisons have been vaccinated (100% coverage). Vaccination of detainees is also rapidly increasing, with only 3.6% refusal rate. Most recent data, obtained in early July, indicate that 84% are fully covered and another 13% have received their first dose, totalling 97% of vaccine coverage. Even though this data does not include Catalonia, as this region is managed under a different administration, it represents the vast majority of Spain, enabling a rough comparison against the vaccination roll-out in the general population. In fact, according to the WHO dashboard, in the same date, the proportion of Spanish citizens living in the community with full coverage represented 34.2% (as opposed to 84.0% in prison) (6).

The strategy adopted in the United Kingdom for prisons has been described as aligned with the general prioritization criteria, which led to some fears given the difficulties to identify people meeting the eligibility criteria because of poor information records (even if better than in most of Europe) (7). However, data obtained from Northern Ireland in June, as one of the five UK nations, indicated a vaccination coverage of 87.3% among the detainees. Also, in Poland, since early July, 74.0% vaccination coverage has been reached among people in prison, considerably higher than reported for population data in the WHO dashboard (44.1% with one dose and 33.5% with full coverage).

There are other countries also progressing, even though not as quickly, but worth highlighting as positive experiences in the region. Finland, Ireland and Sweden have reported, respectively, 34.4, 43.7, and 59.1% coverage amongst detainees in early July. However, whilst in Finland, population coverage is notably higher, with 57.9% having received one dose in early July (and 17.7% fully vaccinated), in Ireland, the roll-out in the general population is going at similar pace as in prison; currently with 49.6% having received one dose (and 32.5% fully vaccinated), and finally, in Sweden vaccination in the general population is considerably lower, currently with 45.8% having received one dose (and 28.9% fully vaccinated). Despite these variations, the general trend supports one of WHO's recommendations on adopting facility-wide vaccination as a more efficient strategy for protecting the vulnerable, with benefits also for surrounding communities (3). These good practice examples are encouraging and are expected to progressively expand in Europe and beyond, leaving no one behind in the pursuit of universal health coverage.

Differences in countries' ability to include people living and working in prison in national COVID-19 vaccination efforts may result from various factors, including political will and financial resource constraints to name a few. Regardless of reasons, the impact of exclusion in terms of social justice, respect for human rights and health outcomes is clear. COVID-19 vaccines are innovative health technologies and, in many ways, an opportunity for societies to advance their health equity commitments by ensuring universal healthcare access for people living in prisons. With this in mind, we highlight the notable efforts of selected Member States to ensure COVID19 vaccination coverage reaches people in prisons. These examples may serve as inspiration to other Member States to follow until equal, fair and universal care is offered to all people deprived of liberty.

## Author Contributions

CF-B conceived the platform through which information is collected and critically revised the dataset created. FA wrote the manuscript and supervised data collection. YA reviewed the literature and provided critical comments to improve the manuscript. All authors reviewed and approved the final version of the manuscript.

## Funding

The Ministry of Social Affairs and Health Finland is the donor for the Health in Prison Programme (annually), although no specific funding was requested for this publication.

## Author Disclaimer

CF-B is staff member of the WHO, FAC and YA are WHO consultants. The authors alone are responsible for the views expressed in this publication and these do not necessarily represent the decisions or the stated policy of the World Health Organization.

## Conflict of Interest

The authors declare that the research was conducted in the absence of any commercial or financial relationships that could be construed as a potential conflict of interest.

## Publisher's Note

All claims expressed in this article are solely those of the authors and do not necessarily represent those of their affiliated organizations, or those of the publisher, the editors and the reviewers. Any product that may be evaluated in this article, or claim that may be made by its manufacturer, is not guaranteed or endorsed by the publisher.
